# The potential impact of co-residence structures on socio-demographic inequalities in COVID-19 mortality

**DOI:** 10.1186/s41118-021-00124-8

**Published:** 2021-09-07

**Authors:** Julien Giorgi, Diederik Boertien

**Affiliations:** 1 National Institute of Statistics and Economic Studies, Montrouge, 92120 France; 2 Center for Demographic Studies, Bellaterra, 08193 Spain

**Keywords:** COVID-19, Demography, Mortality, Social inequalities, Household structure, Education, Nativity, Age

## Abstract

During the COVID-19 pandemic, confinement measures were adopted across the world to limit the spread of the virus. In France, these measures were applied between March 17 and May 10. Using high-quality population census data and focusing on co-residence structures on French territory, this article analyzes how co-residence patterns unevenly put different socio-demographic groups at risk of being infected and dying from COVID-19. The research ambition is to quantify the possible impact of co-residence structures heterogeneity on socio-economic inequalities in mortality stemming from within-household transmission of the virus. Using a simulation approach, the article highlights the existence of theoretical pronounced inequalities of vulnerability to COVID-19 related to cohabitation structures as well as a reversal of the social gradient of vulnerability when the age of the infected person increases. Among young age categories, infection is simulated to lead to more deaths in the less educated or foreign-born populations. Among the older ones, the inverse holds with infections having a greater potential to provoke deaths through the transmission of the virus within households headed by a highly educated or a native-born person. Demographic patterns such as the cohabitation of multiple generations and the survival of both partners of a couple help to explain these results. Even though inter-generational co-residence and large households are more common among the lower educated and foreign born in general, the higher educated are more likely to still live with their partner at higher ages.

## Introduction

In early 2020, the COVID-19 pandemic caused a global health crisis, affecting more than 120 million people in 219 countries and claiming more than 2.7 million lives in a year^1^. Several international studies have already shown how COVID-19 unevenly affects different populations and social groups within countries. In the USA, the over-exposure of African-Americans in particular has been highlighted, pointing out that the health inequalities structuring American society were exacerbated by the pandemic (van Dorn et al. 2020; Yancy 2020) with prevalence rates three times higher for cases and six times higher for deaths in predominantly black counties compared to white ones (Khunti et al. 2020). In general, pre-existing social inequalities in health seem to favor health inequalities in the COVID-19 pandemic as has been shown in China (Chen et al. 2020), Italy (Group and et al 2020), or in Great-Britain (ONS 2020b). Controlling for age, mortality rates in deprived areas of England were more than twice as high as those in privileged areas (ONS 2020a). In France, the number of deaths has increased sharply with marked differences according to the country of birth of the deceased. All causes taken together, deaths in March and April 2020 of people born abroad increased by 48% compared to the same period in 2019 and by 22% for deaths of people born in France (Papon and Robert-Bobée 2020). There is a large variety of possible factors that could underlie these socio-economic differences including living conditions^2^ working conditions,^3^ or pre-existing health inequalities^4^. In this article, we focus on the role that household composition could play in creating socio-economic differences in the risk of dying from COVID-19. By focusing on household structure, we follow earlier research that has shown how demographic factors, such as age, are likely to be important determinants of variation in mortality due to COVID-19 across countries and geographical areas (Dowd et al. 2020;Esteve et al. 2020b;Esteve et al. 2020a;Bajos et al. 2020).

However, the role played by demographic factors is not limited to the age of populations. Demographics can help shed light on the causes of variations in fatality rates by addressing factors such as prevalence of chronic disease, population density, economic disparities, sanitary conditions, and household size and composition (Nepomuceno et al. 2020). Inhabitants of the same dwelling indeed expose each other to the risk of within-household transmission of the virus (Li et al. 2020), and in France, the first results of the EpiCoV survey^5^ suggested the crucial importance of familial transmission with a rate of positive serological tests 6.1 times higher for individuals living with another previously infected person compared to the positivity rate of people living alone (Warszawski et al. 2020). The lockdown measures adopted in France between March and May and again in November and December, designed to limit social interactions and reduce the virus’ reproduction rate, give an even more central role to co-residency structures in the evolution of mortality inequalities in the pandemic caused by the SARS-CoV-2 coronavirus^6^.

By focusing on French data, our main goal is to investigate how co-residence patterns can shape socio-economic inequalities in COVID-19 mortality in France through the channel of within-household transmission. In this work, the inequalities analyzed are the inequalities related to education level, migration status and citizenship status. The education, citizenship, and nativity status are both the benchmarks for many works on inequality, and their study could help to echo international work on COVID-19 social inequalities. The remainder of the article is structured by first presenting a theoretical reflection on differences in household composition across educational levels or nativity status groups that may have an influence on mortality inequalities caused by within-household transmission (“Background” section). At the end of this section, we formulate a set of hypotheses to be tested. The data and the method are presented in a second part (“Data and method” section), followed by the results of our micro-simulation models (“Results” section) and a final discussion.

## Background

In this paper, we aim to study the impact that household arrangements can have on group differences in mortality that can arise from the within-household transmission of COVID-19, even though household arrangements can affect the risk of dying from COVID-19 through other pathways such as the need for external help and the need for contact with members external to the household. Given this focus of the paper on mortality due to the within-household transmission of the virus and the strong age-gradient in case fatality ratios (Verity et al. 2020) two factors become important: the size of households—a limiting factor in the potential number of people to whom the virus can be transmitted within the household following an initial infection—and the age of household members. The more household members, and the older they are, the higher the risk that household members die after a person becomes infected with COVID-19. We label this risk of household members to die from COVID-19 after within-household transmission as vulnerability. We attribute to this vulnerability measure the value of the average number of deaths that an initial infection could trigger within an household through the transmission of the virus to the other household members. In the next section, we discuss how three co-residence types that shape household’s vulnerability are expected to vary by education and migration status: individuals living alone, household size, and multi-generational households.

### Living alone at all ages

The possibility of within-household transmission of COVID-19 is determined by a dichotomous feature of household composition: those made up of several people and those made of people living alone and therefore not at risk of becoming infected by other household members. We here only consider how living alone reduces within-household transmission of COVID-19. Unquestionably, living with relatives provides advantages of mutual assistance or moral support (Arpino et al. 2020), and individuals who live alone might be more at risk of becoming infected by COVID-19 through non-household members, but these aspects are not central to the analysis presented.

An increasing number of people live alone in European countries, and this phenomenon affects all age groups to varying degrees (Esteve et al. 2020c). The age at which one leaves the parental home—which may be linked to economic (Portela and Dezenaire 2014) or cultural (Van de Velde 2008) factors—matters for the share of individuals living alone at young ages. After that, the likelihood of moving in with a partner and having children, then widowhood at older ages, are the main successive causes explaining single-person households (Reher and Requena 2017;Requena et al. 2019). In France, during the first lockdown, 16% of the population lived alone, including a substantial number of people over 75 years of age (Bernard et al. 2020). Through the postponement of household formation and the increase in life expectancy, living alone became more common at all ages before retirement over the last decades (Bodier et al. 2015). After retirement, the proportion of people living alone is much higher and affects women more often, especially after 80 because women are on average younger than their partners, live longer, and re-couple less often after a break-up (Francine et al. 2001).

These average age trends differ by socio-demographic characteristics. Before the age of 45, education is positively correlated with the probability of living alone. Whereas higher educated persons are more likely to live in a couple, lower educated persons are more likely to live with children because of higher rates of single parenthood. After the age of 60, these differences diminish, and after the age of 60, the level of education is associated with a lower probability of living alone. This pattern stems from education-related health inequalities, which reduces the risk of being widowed for higher educated individuals at higher ages as compared to lower educated individuals (Blanpain 2016).

**Hypothesis 1a:** Higher educated individuals have a reduced risk of dying after the within-household transmission of COVID-19 because they are more likely to currently live in a one-person household. However, this negative relationship between vulnerability and education can reverse with age.

Regarding immigration, a stronger link to migration is on average associated with a lower probability of living alone. This might mirror the educational gradient discussed above since immigrants and descendants of immigrants are over-represented in the least educated categories. However, inter-generational cohabitation logic could also explain these figures if there is a higher prevalence of multi-generational households in immigrant populations.

**Hypothesis 1b:** Since people with a migration history are less likely to live alone, migration status should increase vulnerability to dying from COVID-19 after within-household transmission, regardless of the age of the person initially infected.

### Household size

The higher the number of people in a dwelling, the more potential for within-household transmission of COVID-19. Several factors can lead to large families. In France, the number of children is not evenly distributed by level of parental education, with an over-representation of the least educated in large families (Pirus 2004). Step families are also associated with a higher average number of children and are more prevalent among the least educated (Bodier et al. 2015). Immigrants are almost twice more likely to live at home with three or more children than non-immigrants but descendants of immigrants live in almost the same proportions as non-immigrants in households with three or more children and have similar fertility behavior (Blanpain and Lincot 2015).

**Hypothesis 2:** Household size is expected to increase the vulnerability of the least educated populations and those with an immigration background, at all ages.

### Multi-generational households

Since the mortality rate of COVID-19 is highly age-dependent, household size analysis should be accompanied by an analysis in terms of the age of its inhabitants. In particular, the heterogeneous patterns of inter-generational cohabitation can cause high mortality differentials across social groups. Living with one’s parents at different stages of the life cycle can impact inequalities in vulnerability to COVID-19. Education is positively related to the age at first childbearing (Davie 2012). At the same age, the children of parents with higher education can therefore transmit the virus to older people within their households if they still live in the parental home. However, living in the parental home happens less often in more educated households. Among 30-year-olds, lower educated individuals live with their parents three times more often than university graduates. Even after the first departure, it is not uncommon to see people returning to their parents’ home. The reasons put forward in the surveys to explain a return to one’s parents’ home vary according to age, but across all age categories, the loss of employment, financial problems, or health problems account for half of the returns to the parents’ home^7^. These problems predominantly affect individuals with precarious employment status and difficult working conditions, a population in which the least qualified people and foreigners are over-represented.

**Hypothesis 3:** Education and the absence of links with migration reduce vulnerability by lowering the likelihood of living in a multi-generational household.

All of these socially influenced cohabitation patterns suggest that the inequalities in COVID-19 vulnerability linked to within-household transmission depend on multiple factors that may have different directions and significance. Our aim is to give a quantitative indication of the relative importance of these factors in shaping the vulnerability of socio-economic groups to dying from COVID-19 after within-household transmission of the virus.

## Data and method

### Data

The data used are from the Census of the French population carried out by the National Institute of Statistics and Economic Administration (INSEE) from 2009 to 2013. Each year, the census collects information on a subsample of all French households. The French population is divided into five groups, with data on each group being collected from a representative sample of household every 5 years. The inclusion of 5 years of data therefore covers the whole French population. The final sample used in our analysis includes 19.6 million observations representing 31% of the total French population living in private households. Each observation is weighted in order to make the sample representative of the total population in the median year of data collection (2011).

The only selection criteria we used to construct our sample was whether individuals lived in private households. Inhabitants of collective dwellings such as retirement houses were excluded from the sample by the statistical institute providing the data. The data is organized into households and provides information on age, education, citizenship, and migratory status for all individuals in each household. None of the cases was excluded because of missing information on one or more of these variables.

### Measures

#### Decomposing direct and indirect risks

We aim to document group differences in the amount of deaths that are expected to arise after a person becomes infected with COVID-19 and subsequently exposes other household members to infection with the virus. In this case, deaths can be of two types: direct deaths stemming from a “primary” infection when an individual becomes infected outside of the household, and indirect deaths caused by “secondary” infections, i.e., linked to the transmission of the virus from the primarily infected person to other household members. Following the methodology of Esteve and colleagues (Esteve et al. 2020b), we compute for each individual the expected total number of deaths if that person becomes infected with COVID-19. We assume that primary infections occur at random because we concentrate on variation that arises after transmission within the household. Direct deaths per infection equal the age-specific probability of dying once infected and indirect deaths are computed by multiplying the number of co-residents the infected person has in each age category by both the age-specific probability of within-household infection^8^ and the age-specific probability of death following an infection. As the co-residential patterns differ throughout the life cycle, individuals are classified into 10-year age groups.

Formally, we compute the average total number of deaths per infection in a given age category as:

1 $$\begin{array}{*{20}l} Total_{a}&=\frac{\left[\sum_{i=1}^{N_{a}} \left(m_{a}+\sum_{a\in A}n_{i,a}*r_{a}*m_{a}\right)p_{i}\right]}{\sum_{i=1}^{N_{a}}p_{i}}, \forall a \in A \end{array} $$

2 $$\begin{array}{*{20}l}  &=\underbrace{m_{a}}_{\substack{\text{Average direct death} \\ \text{per infection of a person} \\ \text{in age category a}}}+\underbrace{\frac{\sum_{i=1}^{N_{a}}\left(\sum_{a\in A}n_{i,a}*r_{a}*m_{a}\right)p_{i}}{\sum_{i=1}^{N_{a}}p_{i}}}_{\substack{\text{Average indirect deaths through} \\ \text{ within-household transmission} \\ \text{following the infection of a person} \\ \text{in age category a}}} \end{array} $$

with *N*_*a*_ the total number of inhabitants in age category a, *n*_*i*,*a*_ the total number of individual i’s co-resident members in age group *a*∈*A* with *A* the set of all age groups, *p*_*i*_ the individual weight, *r*_*a*_ the age-specific probability of infection computed by Davies et al. (2020),^9^ and *m*_*a*_ the age-specific infection fatality ratios as estimated for 10-year age groups using official epidemiological data in France between 27 May 2020 and 22 February 2021^10^,^11^.

#### Assessing the role of within-household transmission on COVID-19 social inequalities

Since the age-specific probabilities of dying *m*_*a*_ are assumed invariant across social variables but co-residence structures are expected to vary according to the reference level of education or migration status^12^, assessing the role of within-household transmission on inequalities in COVID-19 vulnerability implies averaging the second part of Eq. (2) not only by age but also by education level or nativity status. Formally, we compute the average number of indirect deaths following an infection in a given age category and a given socio-demographic category as:

3 $$\begin{array}{@{}rcl@{}} Index_{a,s_{v}}&=& \left(\frac{\sum_{i=1}^{N_{a,s_{v}}}\left(\sum_{a\in A}n_{i,a,s_{v}}*r_{a}*m_{a}\right)p_{i}}{\sum_{i=1}^{N_{a,s_{v}}}p_{i}}\right) \end{array} $$

with $N_{a,s_{v}}$ the total number of inhabitants in age category a and social category *s* from variable *v*, $n_{i,a,s_{v}}$ the total number of individual i’s co-resident members in age group *a*∈*A* with *A* the set of all age groups, *r*_*a*_ the age-specific probability of infection, and *m*_*a*_ the age-specific infection fatality ratios.

Comparing different values of Eq. (3) for different categories *s* of the same socio-demographic variable *v* identifies how the average differences in co-residence structures between social groups can shape the theoretical inequalities of mortality related to the transmission of the virus within households.

Before moving to the main analysis, we present the socio-demographic variables we use to compare the vulnerability of social groups.

### Head-of-household level variables

The objective is to study the dispersion of the vulnerability index as a function of socio-economic variables, in particular, education, citizenship, and nativity variables. However, not all inhabitants of the same dwelling belong to the same education or migration category. We therefore assign socio-demographic variables at the household level rather than at the individual level using information on the reference person, which is identified in each household following the INSEE methodology^13^. The benefits of this household approach are especially clear for individuals who have not completed their education and still live with their parents. The head-of-household variables *v* from Eq. (3) we use to study socio-economic differences in vulnerability are education, “citizenship status,” and “nativity status.” Educational level has been classified according to the recommendations of the Conference of European Statisticians for the 2010 Population and Housing Censuses and contains 5 levels describing the level of education completed: less than primary, primary, lower secondary, upper secondary, or university completed. Education was preferred to occupational categories because the later did not allow for the inclusion in the analysis of the inactive (including students and retirees) or the unemployed recorded at the time of the cross-sectional data collection. The nativity status distinguishes between individuals born in France and foreign-born individuals whereas the citizenship status is partitioned in three categories: citizen by birth, naturalized citizen, and non-citizen^14^. Information on having parents who migrated to France was not available in the data.

## Results

### Co-residence patterns by age

We start the analysis by describing the household composition of French households and subsequently calculate the connected vulnerability to dying from COVID-19 and how this differs by education, citizenship, and nativity status.

**Fig. 1:**
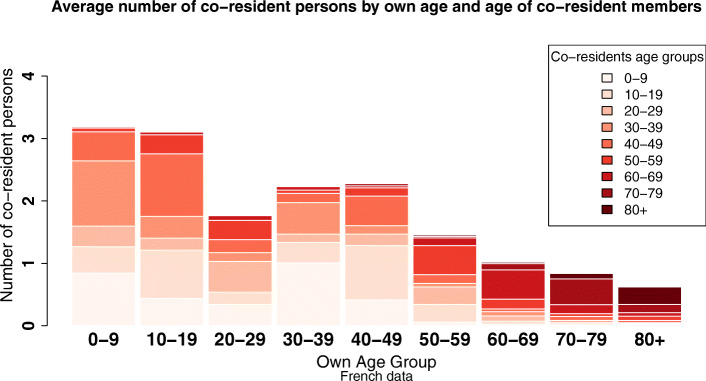
Co-residence structures by age—full sample

Figure 1 shows the average household composition by 10-year age groups. The average number of co-residents ranges from 3.2 for young children to 0.6 for those over 80 years of age (Fig. 1). Children are the populations with the highest average number of co-residents. Between 20 and 29, individuals start leaving the parental home, leading to a fall in the average number of co-residents. Between the ages of 30 and 50, partnering and fertility behaviors increase the average size of households. From the age of 50 onward, children leaving the household coupled with increases in widowhood probability leads to a gradual fall in the number of co-residents. Unlike other age groups, people over 70 essentially live with individuals of their own generation.

### Direct and indirect risks by age

**Fig. 2:**
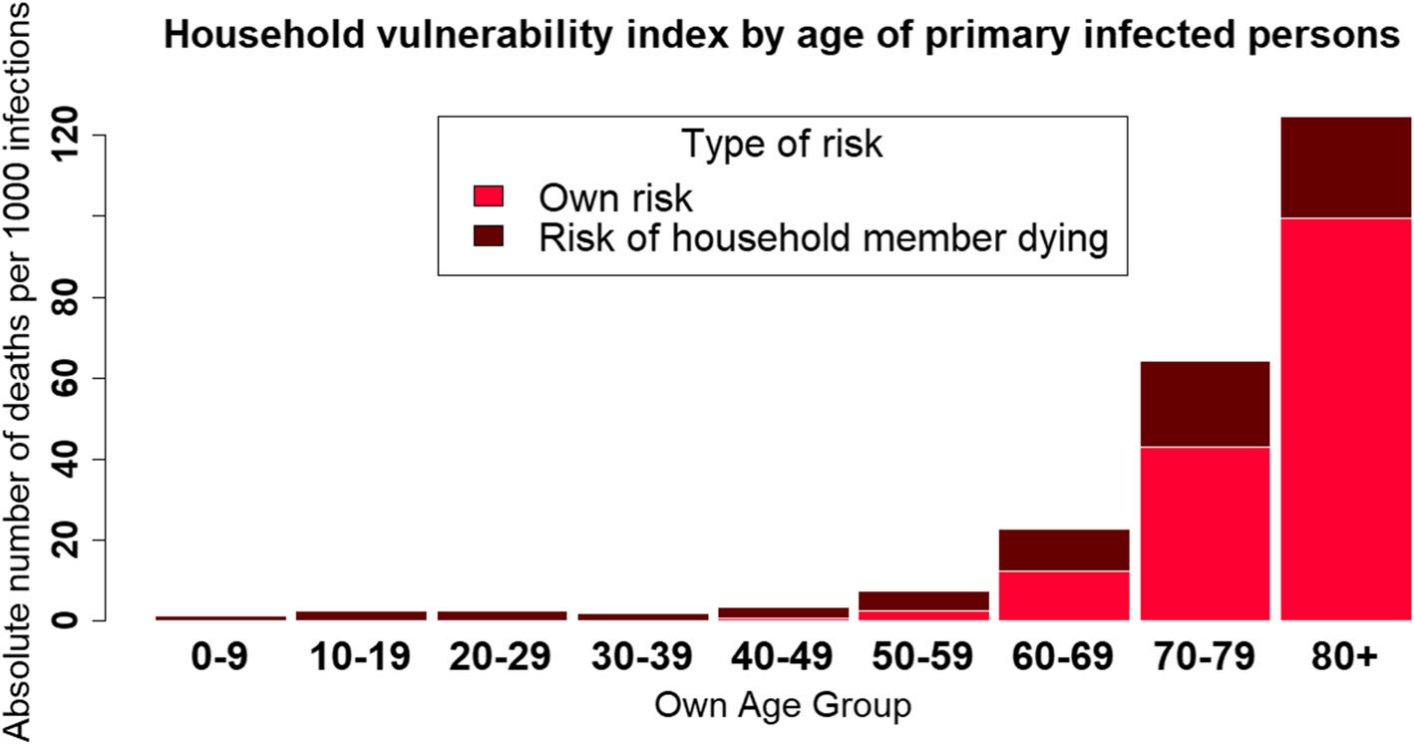
Vulnerability of households to COVID-19-related deaths by age

Figure 2 translates the age differences in household composition into risks of dying from COVID-19. For each age group, the figure indicates the expected number of deaths per 1000 random COVID-19 infections. This graph makes the distinction between “direct” and “indirect” deaths according to the decomposition presented in Eq. (2). The lighter part of the bars shows the age gradient of the fatality case ratio computed with French epidemiological data and relates to “direct” deaths, i.e., the risk that primarily infected persons pass away themselves. The darker part indicates additional deaths transmission of the virus to co-residents could cause. These indirect deaths range from 1.2 deaths per 1000 random infections in the 0–9 years old age category to 25.2 deaths for 1000 primary infections among persons over 80 years of age and 21.4 for the same figure for people between 70 and 79. In other words, the number of deaths that could arise from a person above 70 transmitting the virus to other household members is more than 17 times higher than the number of deaths that children aged 0–9 could cause in that manner. Below 60 years of age, this dispersion is lower. Indirect deaths range from 1.7 deaths per 1000 random infections among 30–39 years old to 5.0 in 50–59 years old. This figure more than doubles to 10.4 indirect deaths for 1000 primary infections among 60–69 years old. These differences are related to the fact that individuals less regularly live with their parents at age 30 and relatively more with children under 10. On the contrary, between 50 and 69, individuals live regularly with their partners since the widowhood rate is relatively low in these age categories and can therefore transmit the virus to a person from the same generation, whose age constitutes a non-negligible COVID-19 mortality risk.

These figures highlight that the vulnerability of a population to the virus not only depends on its age structure (direct deaths), but also on the number and age of other household members to which each individual can transmit the virus (indirect deaths). The significance of within-household transmission on population vulnerability is essential at all ages. The younger the infected individuals are, the greater the indirect deaths are as a proportion of all deaths. Among people under 20, more than 99% of predicted deaths following a primary infection are the deaths of another family member to whom the virus would be transmitted. As age increases, the absolute predicted number of indirect deaths per primary infection increases.

The next section focuses on documenting inequalities in theoretical indirect deaths due to within-household transmission between social groups defined by education category, country of birth, and citizenship status of the head-of-household^15^.

### Education, nativity, and the reversal of the social gradient in mortality over the life cycle

**Fig. 3:**
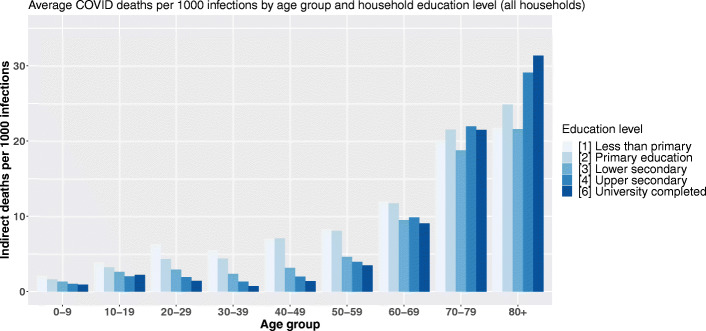
Average COVID-19 indirect deaths per 1000 infections by age group and head-of-household education level

Figure 3 displays the estimated average number of indirect deaths following the infection of 1000 individuals belonging to a given age and head-of-household education level category. In other words, each bar of the graph is a value of $Index_{a,s_{v}} = \left (\frac {\sum _{i=1}^{N_{a,s_{v}}}\left (\sum _{a\in A}n_{i,a,s_{v}}*r_{a}*m_{a}\right)p_{i}}{\sum _{i=1}^{N_{a,s_{v}}}p_{i}}*1000\right)$ with *v* being the reference education level of the household, *s*_*v*_ all the possible education levels, and *a* the 9 age categories. Crossing the age of primarily infected persons with their household’s reference level of education highlights two social gradients: between age groups and between different levels of education within the same age group (Fig. 3).

In the youngest age groups, the lower the household’s reference level of education, the higher the number of indirect deaths following a primary infection. Up to the age of 60, the educative gradient of our COVID-19 demographic vulnerability index is very pronounced within each age group. The greatest dispersion occurs between 30 and 39 years of age, for which persons living in a household with the lowest level of education are estimated to cause 7.5 times more deaths through within-household transmission following their own infection than a person of the same age category in a household where the reference degree is a university degree. As the age of the primarily infected person increases, the education-related vulnerability inequalities shrink and between ages 60 and 69, the head-of-household level of education no longer seems to play a major role. However, after age 70, the direction of our educational gradient in mortality is inverted with our average vulnerability index increasing as the head-of-household education level raises. For instance, in households with a university degree as the reference education level, the infection of an octogenarian will cause significantly more deaths compared to the infection of an octogenarian living in an household with the lowest reference education level. This difference is estimated to be around 44% and to account for an additional 958 deaths per 100,000 primary infections.

**Fig. 4:**
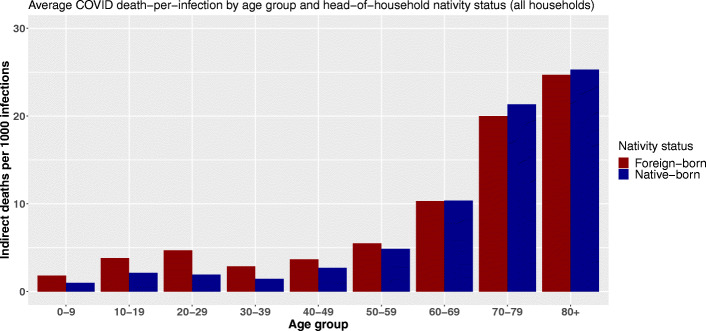
Average COVID-19 indirect deaths per 1000 infections by age group and head-of-household nativity status

Using the birthplace variable, we observe the same shift as the age of primarily infected persons increases (Fig. 4). Following an infection, people living in households headed by a foreign-born person can cause more indirect deaths through within-household transmission up to the age of 60. The relative difference peaks between 20 and 29 years of age. At that age, foreign-born household inhabitants are expected to cause on average 2.4 times more deaths through within-household transmission following an infection. After 30, this ratio shrinks.

From the age of 60 onward, not only does the total number of indirect deaths by infection increase substantially, but inequalities in vulnerability are reversed. The reference population^16^ suffers a comparative disadvantage and experiences a higher average number of indirect deaths per infection. However, these differences remain small, and the reversal of our index gradient is less pronounced than with the level of education and the reversal appears slightly earlier in the life cycle. Estimated mortality differences amount to 60 additional indirect deaths per 100,000 primary infections among octogenarian and 133 among septuagenarians.

**Fig. 5:**
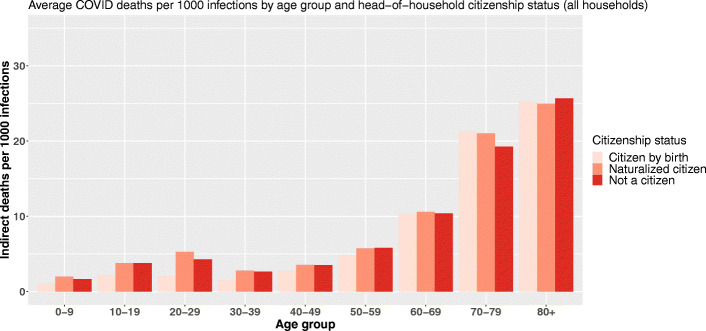
Average COVID-19 indirect deaths per 1000 primary infections by age group and head-of-household citizenship status

Regarding citizenship, the findings are quite similar even if the inversion of the gradient is not that clear (Fig. 5).

Our result that social inequalities in expected COVID-19 mortality related to within-household transmission of the virus are neither of the same magnitude nor the same direction depending on the age of the infected person is interesting and analytically challenging. In the next section, we dive deeper into two questions that emerge from the results:

- Why are the households of the higher educated and native-born less vulnerable under the age of 60?
- Why is it that from the age of 60 onward, inequalities in vulnerability to the virus following an infection seem to diminish and reverse across groups?

### Additional analysis

In the literature review, we discussed previous research to formulate expectations about the prevalence of various household structures among different socio-economic groups and how these could affect vulnerability to COVID-19. In this regard, we discussed the amount of single-person households, household size, and multi-generational households as factors that could cause differences in vulnerability across social groups. Lower educated individuals and individuals with a migration background/non-citizens were expected to live in households that are more vulnerable to within-household transmission-related COVID-19 deaths for various reasons. First of all, they were expected to live in larger households. Table 1 shows the number of household members depending on the educational level, nativity status, and citizenship of the head-of-household. Large households are indeed more prominent among the lower educated, the foreign-born and non-citizens. Second, they were expected to more often live in multi-generational households. Tables 7, 8, 9, 10, 11 and 12 in Appendix C and D, respectively, confirm this expectation, lower educated individuals or individuals with a migration background under the age of 50 living on average with older individuals or with at least one person being one or two generations older. Finally, they were expected to be less likely to live alone during the primary parenting ages. Table 2 shows the proportion of individuals living alone by age, education, nativity status, and citizenship variables. A positive educational gradient in living alone is indeed observed at younger ages, particularly between ages 20 and 39. Similarly, native-born and French citizens are more likely to live alone too.

**Table 1:** Population distribution by head of household variables and household size (full sample, %)

	Population distribution					
Head-of-household variable	**Number of household inhabitants**					
**Education level**	1	2	3	4	5	6+
Less than primary	17.0	28.2	16.9	16.2	11.1	10.6
Primary education	22.4	37.9	15.3	12.9	6.9	4.5
Lower secondary	19.0	32.2	18.9	17.8	8.1	4.2
Upper secondary	12.6	28.2	20.9	23.9	10.2	4.3
University completed	14.6	25.6	19.7	25.4	11.1	3.8
**Citizenship status**	1	2	3	4	5	6+
Citizen by birth	16.1	30.2	19.46	21.6	9.0	3.6
Naturalized citizen	9.5	19.9	17.2	22.3	17.5	13.7
Not a citizen	9.8	20.5	18.8	21.8	15.1	14.0
**Nativity**	1	2	3	4	5	6+
Foreign-born	11.2	22.5	18.2	20.9	14.9	12.4
Native-born	16.0	30.2	19.5	21.8	9.0	3.5

*Note*: 12.6% of individuals living in a household in which the head-of-household has an upper secondary education live alone. 4.3% of them live in households with more than 6 inhabitants

**Table 2:** Proportion of people living alone by age and head-of-household variables

	Proportion of people living alone (%)								
Head-of-household	**Own age**								
**Education level**	0–9	10–19	20–29	30–39	40–49	50–59	60–69	70–79	80+
Less than primary education	0.00	0.3	4.4	8.8	11.8	16.4	22.3	33.9	54.0
Primary education	0.00	0.8	6.8	8.9	11.0	18.7	25.2	34.5	53.6
Lower secondary education	0.00	2.6	10.3	11.1	13.1	20.6	27.9	39.1	57.9
Upper secondary education	0.00	4.3	17.3	11.8	12.3	16.1	19.3	24.4	39.9
University completed	0.00	0.4	28.3	17.1	13.8	18.3	21.9	25.0	37.0
**Citizenship status**	0–9	10–19	20–29	30–39	40-49	50–59	60–69	70–79	80+
Citizen by birth	0.00	2.7	19.7	13.8	13.0	17.9	22.6	31.2	50.5
Naturalized citizen	0.00	0.4	7.6	9.8	8.5	11.8	17.3	25.7	45.9
Not a citizen	0.00	1.3	13.2	11.0	10.4	13.1	16.3	22.7	37.3
**Nativity**	0–9	10–19	20–29	30–39	40–49	50–59	60–69	70–79	80+
Foreign-born	0.00	1.1	11.5	10.8	10.00	14.3	19.2	26.7	45.3
Native-born	0.00	2.7	19.9	13.8	13.1	17.8	22.5	31.2	50.5

*Note*: 9.8% of individuals between 30 and 39 years old and living in a household with a head-of-household being a naturalized citizen live alone. 45.3% of individuals over 80 years old living in a household with a foreign-born head of household live alone

These three factors combined provide explanations for why lower educated individuals; the foreign-born and non-citizens are expected to be at a higher risk of dying from COVID-19 after becoming infected by a household member. However, these gradients in vulnerability reverse at later ages. For education, the clearest explanation can be found in the number of persons living alone. Table 2 shows that after age 60, the higher educated are less likely to live alone than lower educated persons. To estimate to what extent these differences in the likelihood of living in a single-person household can explain our results, we restrict the analysis to households of more than two persons in additional analysis (Appendix Figs. 15, 16, and 17 and Tables 13 and 15). For the education variable, the reversal of the gradient indeed disappears and the gaps beyond the age of 70 turn into a disadvantage for the least educated categories. A parametric model estimated for households with more than two persons is consistent with these results (Appendix G—Tables 16 and 17). These models also confirm for nativity status and citizenship that differences in living in a single-person household cannot explain why the gradient in vulnerability reverses at later ages.

Additional analysis (Appendix C, D, and E) shows that the main reason is that even though older persons who are not born in France or not a citizen live in larger households and are less likely to live alone, they live with fewer persons who are at a high risk of dying from COVID-19. In other words, the age composition of these households is different. Whereas native-born and citizens predominantly live with their partner or alone at later ages, the foreign-born, and non-citizens less often live with a person from their own generation i.e. their partner^17^.

#### Robustness and sensitivity tests

Our results are consistent with detailed demographic explanations. However, our microsimulation model relies on the assumptions that may shape our results.

Indeed, our modeling first assumes heterogeneity in the probability of being infected with the virus as a function of age. This assumption and the probabilities of infection incorporated into the model are based on the work of Davies and colleagues (Davies et al. 2020). Setting the probability of transmission to the other inhabitants of the dwelling to 1 for all individuals allows to give an upper-bound limit of the number of deaths that could result from an infection and to check the sensitivity of our results to this age-specific infection rate hypothesis. Figures 12, 13, and 14 available in Appendix F.2 synthesize the results of these models and show that our results—and, in particular, the reversal of mortality inequalities with the age of primarily infected persons—are robust to changes in the transmission rate.

In addition, the age-specific mortality rates are pivotal in shaping the results. We made the methodological choice to apply to our model the mortality rates computed with comprehensive French epidemiological data. However, the robustness of our results to a change in these mortality rates was tested by using the age-specific mortality rates calculated byVerity et al. (2020) and used for example in the work of Esteve and colleagues (Esteve et al. 2020b;Esteve et al. 2020a). The results obtained with these new mortality rates are available in Appendix F.3 and are also consistent with the model used in the article. Only the inequalities observed between native and foreign-born for primary infection at older ages are slightly smaller.

Finally, our model has been designed to separately address inequalities by education, birthplace and citizenship status. We provide in the Appendix the details of a simple parametric model to control for the robustness of our results by controlling jointly—rather than separately—for education and birthplace category. This model also includes a set of additional other demographic variables and is reproduced for different household sizes (Appendix F.4).

## Discussion

The aim of this article was to investigate to what extent differences in co-residence structures could cause variation in deaths through the within-household transmission of COVID-19 across social groups. Previous research has shown that mortality related to COVID-19 is socially stratified. Among the possible reasons that those from disadvantaged social groups are more likely to die from COVID-19 are differences in co-residence structures. In this article, we investigated to what extent this could be the case by relying on a simulation exercise.If a random person becomes infected with COVID-19, how many individuals are expected to die from the transmission of the virus to this person’s household members? We found that education, citizenship, and place of birth of the head of household are all related to this expected number of deaths. The simulations showed that the infection of lower educated individuals, of persons who were born abroad, and of non-citizens are indeed expected to lead to more deaths related to within household transmission of the virus as compared the infection of higher educated individuals, the native born and citizens. However, an interesting element that came forward in the analysis is that these socio-economic gradients in vulnerability reverse with age. At higher ages, higher educated individuals, the native born, and citizens are more likely to still live with their partner. Given that the partners of older individuals are at higher risk of dying after infection with COVID-19, this increases the risk that someone dies after a higher educated, native-born, or citizen becomes infected with COVID-19. There are important limitations that have to be taken into account when interpreting this result. Firstly, the current work does not allow including the co-morbidity factors between the different socio-demographic categories studied. It is possible that case fatality ratios at higher ages differ by education due to comorbidity, a refinement we could not take into account. Secondly, collective housing information was not available either, an issue we have to leave for future research. Thirdly, in addition to demographic data, the calibration of the model was enriched with epidemiological data concerning the probability of virus transmission and mortality by age group. The likelihood of transmission probably differs according to other variables too. Future work could look at weighting the likelihood of virus transmission by type of family, intimate or social link between each pair of inhabitants within the same household. Fourthly, we did not take into account possible differences by gender. Women might more often live with household members (children/parents) and therewith live in more vulnerable households, but at later ages, women might be more likely to live alone as they are more likely to survive their partner. Future research can look into this further. Finally, it has to be emphasized that our analysis relies on simulations based on estimated case fatality ratios. The extent to which within-household transmission-related mortality varies across social groups in reality is a question that only future data collections can answer. Nonetheless, our results do confirm the concerns that socio-eonomically disadvantaged individuals are more likely to live in households vulnerable to COVID-19. However, this conclusion only holds under the age of 60. At later ages, co-residence structures change and socio-economically advantaged individuals are more likely to live with persons at high risk of dying from COVID-19. This could be taken into account when interpreting observed socio-economic gradients in mortality.

## Conclusion

The role of cohabitation structures and their heterogeneity by age and social group play an important role in the theoretical social inequalities in COVID-19 mortality. By measuring vulnerability inequalities as the theoretical differences in deaths caused by the virus through within-household transmission following a random initial infection, the results highlight that these inequalities are important regardless of the age of person initially infected. However, the direction of the differences in average indirect deaths per infection change with the age of the initially infected person. The number of indirect deaths by infection is higher in the least educated households or those with a migration background in the youngest age categories. Then, these figures balance out before turning to the disadvantage of domestic-born or more educated populations for initial infections of individuals over the age of 60 years. These results—underlining the fact that cohabitation patterns alone can be a tenfold element of pronounced social inequalities in mortality—find a strong resonance at a time when the return to lockdown measures is re-investing public debate as the third wave of epidemics appears in France. Also, by decomposing vulnerability inequalities on the basis of two criteria—namely the age and socio-demographic category of the primarily infected person—we posit that the social gradient in COVID-19 vulnerability linked to within-household transmission is subject to finer logic than the social gradient in mortality usually presented in the literature as its direction changes with the age of the initially infected person. The same pattern is observed for education level and place of birth, but a comparison of the age-specific cohabitation logics of these two variables shows that the underlying logics are different after the age of 60. For the level of education, single-person households are the main factor in inequalities, whereas in the case of foreign-born individuals, households composed of several generations and lower widowhood inequalities with domestic-born persons reduce the observed inequalities.

## Appendix

## A. Descriptive statistics of the sample

**Table 3:** Distribution of individuals according to their head-of-household variables (full sample, %)

Head-of-household	
**Education Level**	Share
Less than primary	13%
Primary	12%
Lower secondary	5%
Upper secondary	43%
University completed	27%
**Citizenship status**	Share
Citizen by birth	86.4%
Naturalized citizen	6.3%
Not a citizen	7.3%
**Nativity status**	Share
Foreign-born	17%
Native-born	83%

*Note*: 27% of individuals live in an household in which the head of household has a university degree. 83% of individuals live in a household in which the head-of-household was born in France

## B. Comparison tables between individual and head-of-household variables

**Table 4:** Comparison table between individual and head-of-household variable citizenship status

	Head-of-household level		
**Individual level**	Citizen by birth	Naturalized citizen	Not a citizen
Citizen by birth	0.95	0.03	0.02
Naturalized citizen	0.18	0.71	0.11
Not a citizen	0.10	0.05	0.85

*Note*: 95% of citizen by birth live in an household in which the head of household is a citizen by birth. 2% of them live in a household in which the head-of-household is not a citizen

**Table 5:** Comparison table between individual and head-of-household variable education level

	Head-of-household level				
**Individual level**	Less than primary	Lower secondary	Primary	University completed	Upper secondary
NIU (not in universe)	0.11	0.04	0.07	0.32	0.46
Less than primary	0.74	0.02	0.06	0.04	0.15
Primary	0.07	0.02	0.71	0.03	0.16
Lower secondary	0.07	0.38	0.07	0.15	0.32
University completed	0.02	0.01	0.02	0.81	0.14
Upper secondary	0.05	0.02	0.04	0.07	0.81

**Table 6:** Comparison table between individual and head-of-household variable nativity status

	Head-of-household level	
**Individual level**	Foreign-born	Native-born
Foreign-born	0.84	0.16
Native-born	0.08	0.92

## C. Share of co-residents members by age

**Table 7:** Share of co-residents members by age and head-of-household citizenship status

	Co-residents age (%)								
Head-of-household variable	**Citizen by birth**								
**Own age**	0–9	10–19	20–29	30–39	40–49	50–59	60–69	70–79	80+
0–9	25.60	12.80	10.20	34.50	14.50	1.70	0.50	0.20	0.10
10-19	13.30	24.40	5.40	11.40	34.20	9.70	1.10	0.30	0.20
20-29	19.50	9.90	28.60	7.80	12.00	17.70	3.60	0.60	0.30
30–39	45.20	14.40	5.30	23.40	6.40	2.20	2.20	0.60	0.20
40–49	17.10	38.90	7.40	5.80	21.60	5.80	1.40	1.40	0.50
50–59	3.30	18.50	18.50	3.30	9.80	34.60	8.60	1.50	1.80
60–69	1.80	3.70	6.70	6.00	4.30	15.50	49.50	10.00	2.60
70–79	1.10	1.90	1.90	3.10	7.50	4.80	17.20	51.60	11.10
80+	1.00	2.00	2.10	1.70	5.60	11.20	8.70	21.60	46.20
Head-of-household variable	**Not a citizen**								
**Own age**	0–9	10–19	20–29	30–39	40–49	50–59	60–69	70–79	80+
0–9	29.30	14.90	11.90	26.10	13.50	2.80	1.00	0.30	0.10
10–19	17.40	25.90	10.50	11.00	21.90	9.70	2.70	0.70	0.20
20–29	18.90	14.30	26.00	9.50	9.90	13.30	6.30	1.50	0.30
30–39	41.50	14.90	9.50	18.80	7.20	3.30	3.30	1.30	0.20
40–49	23.40	32.40	10.80	7.80	16.00	5.40	2.00	1.60	0.50
50–59	8.00	23.50	23.80	5.90	8.90	18.40	8.30	2.00	1.10
60–69	4.90	11.00	19.00	9.90	5.60	14.00	26.10	8.10	1.50
70–79	3.70	6.80	10.30	8.50	10.00	7.80	18.40	28.10	6.40
80+	3.10	5.00	6.10	4.70	10.10	13.70	10.80	20.50	25.80
Head-of-household variable	**Naturalized citizen**								
**Own age**	0–9	10–19	20–29	30–39	40–49	50–59	60–69	70–79	80+
0–9	28.50	16.10	8.40	25.90	16.10	3.30	1.10	0.40	0.10
10–19	16.00	26.20	9.20	8.90	24.90	11.60	2.20	0.70	0.30
20–29	15.10	16.70	20.80	7.20	12.10	19.90	6.60	1.20	0.40
30–39	44.60	15.50	6.90	16.80	7.70	3.80	3.40	1.10	0.20
40–49	23.30	36.00	9.80	6.40	14.90	5.90	1.80	1.50	0.50
50–59	7.20	25.50	24.10	4.80	8.90	19.40	7.10	1.60	1.40
60–69	5.10	10.40	17.20	9.10	5.70	15.00	27.40	7.90	2.10
70–79	4.10	6.50	6.60	6.30	10.00	7.10	16.60	33.60	9.30
80+	2.80	5.30	4.80	3.00	7.50	13.10	9.40	19.70	34.50

*Note*: 45.2% of the people with whom people aged 30 to 39 in a household headed by a citizen by birth live are children aged 0 to 9

**Table 8:** Share of co-residents members by age and head-of-household education level

	Co-residents age (%)								
Head-of-household variable	**Less than primary education**								
**Own age**	0–9	10–19	20–29	30–39	40–49	50–59	60–69	70–79	80+
0–9	27.80	19.80	12.60	21.60	13.00	3.30	1.20	0.50	0.10
10–19	16.50	26.40	10.20	11.10	22.50	9.80	2.60	0.80	0.30
20–29	16.70	16.30	20.60	6.40	12.70	17.80	7.20	1.80	0.50
30–39	34.50	21.20	7.70	14.80	7.50	5.40	5.80	2.60	0.50
40–49	16.40	34.20	12.10	6.00	16.20	6.60	3.00	3.90	1.50
50–59	5.60	19.70	22.40	5.60	8.70	22.70	8.90	3.20	3.20
60–69	3.20	7.90	13.90	9.30	6.10	13.70	31.00	11.50	3.40
70–79	1.50	3.30	4.60	5.50	10.50	6.50	15.20	41.40	11.40
80+	0.90	2.10	2.40	2.20	7.90	13.00	9.00	22.60	39.90
Head-of-household variable	**Primary education**								
**Own age**	0–9	10–19	20–29	30–39	40–49	50–59	60–69	70–79	80+
0–9	27.00	16.20	13.90	25.80	12.90	2.70	1.00	0.40	0.10
10–19	14.80	24.60	7.90	12.10	27.10	10.60	2.00	0.60	0.30
20–29	20.00	12.40	20.40	6.50	13.10	19.90	5.90	1.10	0.50
30–39	37.30	19.10	6.50	16.70	7.00	4.90	5.90	2.00	0.40
40–49	15.00	34.50	10.60	5.70	18.10	6.90	3.40	4.30	1.60
50–59	3.70	15.60	18.70	4.60	8.10	30.80	11.30	3.10	4.10
60–69	1.70	3.60	6.60	6.60	4.80	13.50	46.00	13.20	4.00
70–79	0.70	1.30	1.50	2.70	7.20	4.50	16.00	52.20	13.90
80+	0.40	0.90	1.10	1.00	4.30	9.50	7.80	22.40	52.60
Head-of-household variable	**Lower secondary education**								
**Own age**	0–9	10–19	20–29	30–39	40–49	50–59	60–69	70–79	80+
0–9	25.90	14.90	14.30	27.70	13.40	2.70	0.70	0.20	0.10
10–19	13.00	24.30	7.20	10.80	30.60	11.90	1.40	0.40	0.20
20–29	20.40	11.70	22.90	6.00	13.20	20.70	4.10	0.70	0.40
30–39	41.30	18.30	6.30	18.70	6.80	4.00	3.50	0.90	0.30
40–49	14.70	38.20	10.10	5.00	20.00	7.90	1.80	1.60	0.60
50–59	3.60	18.30	19.60	3.60	9.70	33.60	8.20	1.50	1.80
60–69	1.90	4.10	7.30	5.90	4.20	15.60	49.00	9.30	2.70
70–79	1.10	2.20	2.20	2.80	7.00	5.30	17.00	51.20	11.20
80+	1.00	2.30	2.20	1.60	4.90	11.40	8.90	20.20	47.40
Head-of-household variable	**Upper secondary education**								
**Own age**	0–9	10–19	20–29	30–39	40–49	50–59	60–69	70–79	80+
0–9	25.20	13.10	11.90	33.30	14.00	1.70	0.50	0.20	0.10
10–19	13.40	23.90	5.70	12.50	34.40	8.70	0.90	0.30	0.20
20–29	21.70	10.10	26.90	7.80	13.10	16.80	2.90	0.50	0.30
30–39	44.60	16.50	5.70	21.70	6.90	2.20	1.90	0.40	0.10
40–49	16.30	39.20	8.40	6.00	22.00	5.90	1.10	0.90	0.30
50–59	3.50	17.70	19.10	3.40	10.50	35.60	8.10	1.00	1.10
60–69	2.20	3.80	6.80	6.00	4.10	16.50	50.50	8.50	1.70
70–79	1.70	2.70	2.40	3.10	7.00	4.70	18.60	51.80	8.00
80+	1.90	3.60	3.60	2.50	6.50	12.80	9.50	20.30	39.40
Head-of-household variable	**Unversity degree**								
**OWN AGE**	0–9	10–19	20–29	30–39	40–49	50–59	60–69	70–79	80+
0–9	26.90	10.30	5.80	38.60	16.30	1.50	0.40	0.10	0.10
10–19	13.40	25.10	4.30	8.50	35.90	11.30	1.10	0.20	0.10
20–29	15.00	8.50	38.00	10.40	7.50	16.10	3.80	0.40	0.30
30–39	50.40	8.50	5.20	27.60	5.70	1.10	1.10	0.30	0.10
40–49	23.30	39.10	4.10	6.30	21.10	4.60	0.80	0.50	0.20
50–59	4.30	25.20	18.20	2.40	9.40	31.30	7.50	0.80	0.80
60–69	2.40	5.20	9.10	5.50	3.60	16.00	49.40	7.20	1.50
70–79	2.40	3.30	2.90	3.60	6.00	5.00	20.30	47.70	8.90
80+	2.20	3.80	3.80	2.60	5.20	10.40	9.60	19.60	42.80

*Note*: 34.5% of the people with whom people aged 30 to 39 in a household headed by a person with primary education live are children aged 0 to 9

**Table 9:** Share of co-residents members by age and head-of-household nativity status

	Co-residents age (%)								
Head-of-household variable	**Native-born**								
**Own age**	0–9	10–19	20–29	30–39	40–49	50–59	60–69	70–79	80+
0–9	25.60	12.70	10.30	34.90	14.40	1.50	0.50	0.20	0.10
10–19	13.40	24.30	5.20	11.70	34.60	9.30	1.00	0.30	0.20
20–29	20.00	9.70	29.00	7.90	12.10	17.10	3.40	0.50	0.30
30–39	45.40	14.40	5.30	23.60	6.40	2.10	2.10	0.60	0.20
40–49	17.10	38.90	7.40	5.80	21.80	5.70	1.40	1.40	0.50
50–59	3.20	18.30	18.20	3.30	9.90	35.00	8.60	1.50	1.90
60–69	1.80	3.50	6.40	6.00	4.20	15.40	50.10	10.00	2.60
70–79	1.10	1.90	1.80	3.00	7.50	4.70	17.00	52.10	11.10
80+	0.90	1.90	2.00	1.70	5.60	11.10	8.50	21.60	46.60
Head-of-household variable	**Foreign-born**								
**Own age**	0–9	10–19	20–29	30–39	40–49	50–59	60–69	70–79	80+
0–9	28.70	15.70	10.10	25.60	15.10	3.30	1.10	0.40	0.10
10–19	16.10	25.90	9.80	9.40	23.60	11.80	2.50	0.70	0.20
20–29	16.20	15.30	23.50	7.90	10.80	17.90	6.60	1.30	0.40
30–39	42.50	15.10	8.20	17.70	7.40	3.90	3.70	1.30	0.20
40–49	22.70	34.50	10.20	6.80	15.40	6.20	2.10	1.60	0.60
50–59	6.90	24.00	23.50	4.90	8.70	21.10	7.80	1.70	1.30
60–69	4.30	9.70	16.60	8.80	5.50	14.80	30.00	8.30	2.00
70–79	3.30	5.90	7.40	6.60	9.50	7.30	18.30	33.10	8.50
80+	2.40	4.60	4.70	3.10	7.80	13.20	10.50	20.40	33.30

*Note*: 45.4% of the people with whom people aged 30 to 39 in a household headed by a native-born person live are children aged 0 to 9

## D. Proportion of individuals with at least one co-resident by age-category

**Table 10:** Proportion of individuals with at least one co-resident by age and head-of-household citizenship status

Proportion of indivduals with at least one co-resident by age category									
	**Co-residents age**								
Head-of-household variable	**Citizen by birth**								
**Own age**	0–9	10–19	20–29	30–39	40–49	50–59	60–69	70–79	80+
0–9	60.30	30.30	22.40	61.00	28.00	3.50	1.30	0.60	0.40
10–19	28.40	55.60	12.60	22.30	55.40	19.50	2.70	1.20	0.90
20–29	23.50	14.00	43.20	11.20	14.20	21.00	5.50	1.30	1.10
30–39	69.90	26.00	12.30	50.80	12.70	4.20	5.20	2.20	0.90
40–49	33.40	69.00	14.60	13.90	47.60	13.40	3.80	5.30	2.90
50–59	4.50	22.80	20.50	3.90	12.70	47.30	14.70	3.60	5.80
60–69	1.30	2.90	4.80	3.70	2.70	11.70	47.30	13.60	4.50
70–79	0.50	0.90	0.80	1.20	2.60	1.90	9.50	41.10	12.80
80+	0.20	0.50	0.50	0.40	1.10	2.30	2.30	8.80	27.50
Head-of-household variable	**Not a citizen**								
**Own age**	0–9	10–19	20–29	30–39	40–49	50–59	60–69	70–79	80+
0–9	68.20	42.70	29.60	59.20	39.20	10.90	4.80	2.90	1.90
10–19	33.10	63.00	22.80	24.30	51.30	31.30	11.10	5.50	3.20
20–29	32.40	27.60	48.10	20.10	21.30	33.40	19.60	8.60	4.20
30–39	68.10	32.60	21.20	44.50	19.60	10.60	12.50	8.70	3.80
40–49	39.00	61.90	19.50	17.20	41.50	17.80	7.80	10.40	8.20
50–59	9.20	31.10	26.40	7.20	14.20	37.30	21.10	8.90	11.10
60–69	3.30	9.50	13.50	6.70	4.90	17.00	39.50	22.00	9.10
70–79	1.10	2.60	3.50	2.80	3.60	4.00	12.10	33.80	19.20
80+	0.30	0.60	0.70	0.50	1.20	2.10	2.20	7.70	24.20
Head-of-household variable	**Naturalized citizen**								
**Own age**	0–9	10–19	20–29	30–39	40–49	50–59	60–69	70–79	80+
0–9	69.60	40.60	27.00	65.00	42.00	10.50	4.80	2.80	1.30
10–19	36.80	65.30	31.00	26.80	59.50	35.00	10.10	4.60	2.50
20–29	24.40	25.40	44.60	16.00	21.20	35.80	17.80	5.20	2.80
30–39	68.20	27.10	18.50	43.60	17.90	9.00	10.90	5.80	2.00
40–49	46.50	68.10	27.30	20.00	41.50	18.40	7.50	9.10	4.90
50–59	11.00	35.90	42.20	8.60	16.50	40.40	21.30	7.10	8.40
60–69	3.60	7.70	16.00	7.30	4.60	14.70	39.00	17.80	6.40
70–79	1.40	2.30	3.10	2.60	3.60	3.10	11.20	36.00	14.70
80+	0.50	0.90	1.10	0.60	1.40	2.60	2.90	9.90	25.90

*Note*: 60.3% of people aged 0–9 living in a household with a citizen by birth head of household live at least with another person aged beween 0 and 9

**Table 11:** Proportion of individuals with at least one co-resident by age and head-of-household education level

Proportion of indivduals with at least one co-resident by age category									
	**Co-residents age**								
Head-of-household variable	**Less than primary education**								
**Own age**	0–9	10–19	20–29	30–39	40–49	50–59	60–69	70–79	80+
0–9	66.00	39.50	30.00	53.90	27.00	6.50	2.60	0.90	0.40
10–19	44.30	62.70	31.50	35.80	50.60	23.20	6.60	2.00	0.80
20–29	35.70	27.30	45.70	17.40	22.70	27.90	12.20	2.90	1.10
30–39	61.30	32.40	16.80	38.20	13.80	8.50	9.70	4.20	1.10
40–49	39.90	62.10	29.50	19.70	38.50	14.50	6.80	8.00	4.10
50–59	11.30	30.40	41.20	12.20	15.80	38.40	16.50	5.20	6.70
60–69	4.20	8.70	18.30	12.70	6.40	15.10	37.40	13.00	4.70
70–79	1.50	2.80	4.90	6.10	8.00	5.00	13.80	35.40	12.80
80+	0.50	0.90	1.30	1.30	3.30	5.00	3.90	9.70	22.80
Head-of-household variable	**Primary education**								
**Own age**	0–9	10–19	20–29	30–39	40–49	50–59	60–69	70–79	80+
0–9	62.50	34.00	30.10	55.40	24.10	3.70	1.10	0.40	0.20
10–19	35.80	57.00	21.20	31.00	49.60	16.10	2.60	0.80	0.40
20–29	35.30	20.90	41.30	14.70	19.70	20.60	5.20	0.90	0.50
30–39	62.50	30.90	14.20	40.30	12.50	5.80	5.60	1.90	0.50
40–49	34.40	63.40	23.40	17.10	40.20	10.90	4.20	4.90	2.10
50–59	8.00	27.90	34.40	10.00	15.40	41.80	12.60	3.20	4.60
60–69	3.00	5.90	11.60	11.40	6.60	15.30	43.10	11.90	3.70
70–79	1.10	1.80	2.30	4.20	7.90	3.90	12.30	39.00	11.70
80+	0.40	0.80	1.10	1.00	3.20	5.00	3.50	10.30	27.70
Head-of-household variable	**Lower secondary education**								
**Own age**	0–9	10–19	20–29	30–39	40–49	50–59	60–69	70–79	80+
0–9	59.40	28.50	27.80	59.30	23.30	3.70	1.20	0.60	0.40
10-19	32.10	54.00	18.00	29.00	53.80	18.80	2.80	1.20	0.90
20–29	33.70	18.10	42.10	13.70	18.40	21.40	5.50	1.30	1.00
30–39	61.40	25.10	11.60	43.00	10.60	4.50	4.80	1.80	0.70
40–49	32.80	64.30	20.60	15.70	42.70	12.80	3.60	4.50	2.20
50–59	7.50	28.50	30.40	7.80	16.70	44.60	13.90	3.40	5.00
60–69	2.00	3.80	7.00	6.30	3.50	10.90	43.90	11.90	3.90
70–79	0.70	1.10	1.20	1.80	3.00	1.90	8.30	36.00	10.10
80+	0.40	0.70	0.70	0.60	1.20	2.20	2.30	7.80	23.80
Head-of-household variable	**Upper secondary education**								
**Own age**	0–9	10–19	20–29	30–39	40–49	50–59	60–69	70–79	80+
0–9	59.60	30.00	26.40	63.70	27.10	3.80	1.60	1.10	1.10
10–19	29.60	54.30	13.80	26.90	57.10	19.20	2.90	1.80	2.10
20–29	27.50	14.50	43.70	12.70	16.10	22.20	5.80	1.80	2.30
30–39	69.50	28.00	13.20	49.60	13.30	4.40	5.30	2.40	1.80
40–49	33.00	68.40	16.80	15.80	49.00	14.70	3.80	5.40	4.30
50–59	4.70	20.50	20.80	4.10	13.10	49.80	16.50	3.80	8.10
60–69	1.40	2.40	4.30	3.30	2.20	11.30	50.60	16.50	6.10
70–79	0.50	0.80	0.70	0.90	1.60	1.40	8.50	46.30	15.80
80+	0.20	0.40	0.50	0.30	0.70	1.50	1.60	7.10	30.90
Head-of-household variable	**Unversity degree**								
**Own age**	0–9	10–19	20–29	30–39	40–49	50–59	60–69	70–79	80+
0–9	63.60	32.10	14.60	60.90	37.80	4.90	1.80	1.50	1.20
10–19	23.20	59.60	8.40	13.10	56.20	27.20	4.00	2.20	2.10
20–29	13.60	11.30	44.00	10.10	8.40	21.90	7.60	2.20	2.50
30–39	74.60	20.20	13.00	55.00	14.50	3.40	4.80	2.80	1.70
40–49	36.40	73.80	7.50	11.40	48.90	14.00	3.40	4.70	3.60
50–59	4.10	27.00	14.50	1.90	10.60	46.60	15.80	4.10	7.00
60–69	1.00	3.10	4.00	1.80	1.80	11.10	49.00	18.10	6.50
70–79	0.40	0.70	0.50	0.50	1.00	1.20	7.10	42.50	15.50
80+	0.20	0.40	0.30	0.20	0.40	1.10	1.50	7.80	34.10

*Note*: 60.3% of people aged 0–9 living in a household with a citizen by birth head of household live at least with another person aged beween 0 and 9

**Table 12:** Proportion of individuals with at least one co-resident by age and head-of-household nativity status

Proportion of indivduals with at least one co-resident by age category									
	**CO-RESIDENTS AGE**								
Head-of-household variable	**Native-born**								
**Own age**	0–9	10–19	20–29	30–39	40–49	50–59	60–69	70–79	80+
0–9	60.30	30.30	22.70	61.20	27.90	3.40	1.30	0.60	0.40
10–19	28.10	55.60	12.20	22.30	55.50	19.20	2.60	1.10	0.90
20–29	23.50	13.60	43.40	11.10	14.10	20.70	5.30	1.20	1.00
30–39	70.20	26.40	12.50	51.20	12.80	4.20	5.10	2.20	0.90
40–49	33.00	69.40	14.40	13.80	48.00	13.40	3.80	5.30	2.90
50–59	4.20	21.80	19.60	3.70	12.50	47.50	14.60	3.50	5.80
60–69	1.20	2.70	4.50	3.50	2.60	11.70	47.70	13.50	4.40
70–79	0.50	0.80	0.80	1.20	2.60	1.90	9.50	41.40	12.70
80+	0.20	0.50	0.50	0.40	1.10	2.30	2.30	8.80	27.60
Head-of-household variable	**Foreign-born**								
**Own age**	0–9	10–19	20–29	30–39	40–49	50–59	60–69	70–79	80+
0–9	67.90	39.40	26.30	60.40	38.80	9.10	3.80	2.30	1.20
10–19	35.30	62.80	25.70	25.00	54.80	30.80	8.90	4.20	2.30
20–29	28.30	26.10	45.40	18.00	20.80	32.10	15.90	5.60	2.70
30–39	67.00	27.60	18.30	42.90	17.40	8.40	10.00	6.00	2.10
40–49	43.10	64.20	22.00	18.30	40.30	16.40	6.70	8.60	5.10
50–59	10.80	35.60	34.70	8.50	16.40	40.10	19.70	7.20	8.60
60–69	3.50	8.50	14.50	7.50	5.00	14.90	40.00	19.10	7.00
70–79	1.20	2.40	3.20	2.80	3.70	3.20	11.00	34.60	15.30
80+	0.40	0.80	0.90	0.60	1.40	2.30	2.50	8.90	25.10

*Note*: 27.6% of people aged 80+ living in a household with a native-born head of household live at least with another person aged over 80

## E. Households structures by head-of-household variables

**Fig. 6:**
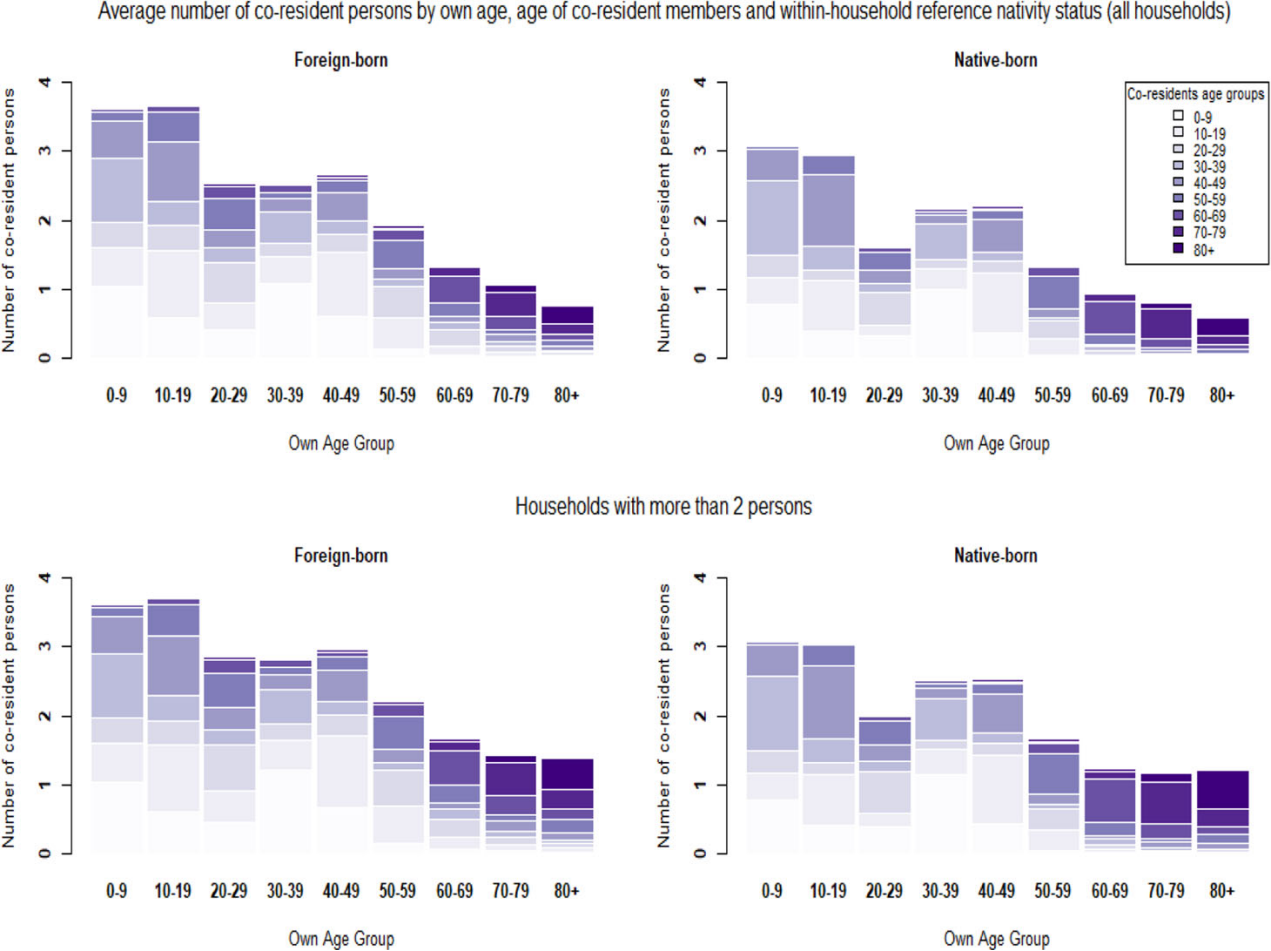
Household structures by head-of-household nativity status

**Fig. 7:**
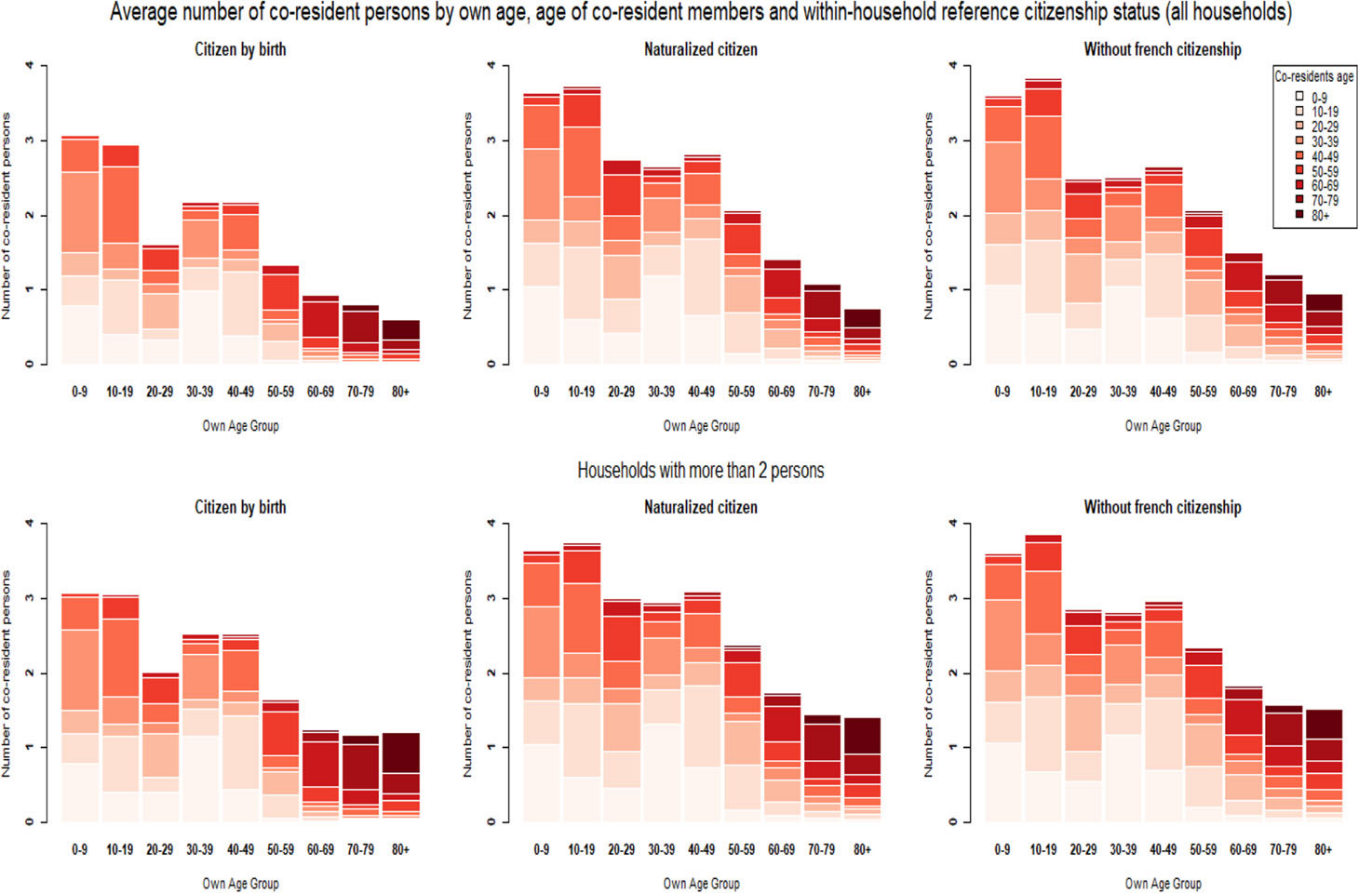
Household structures by head-of-household citizenship status

**Fig. 8:**
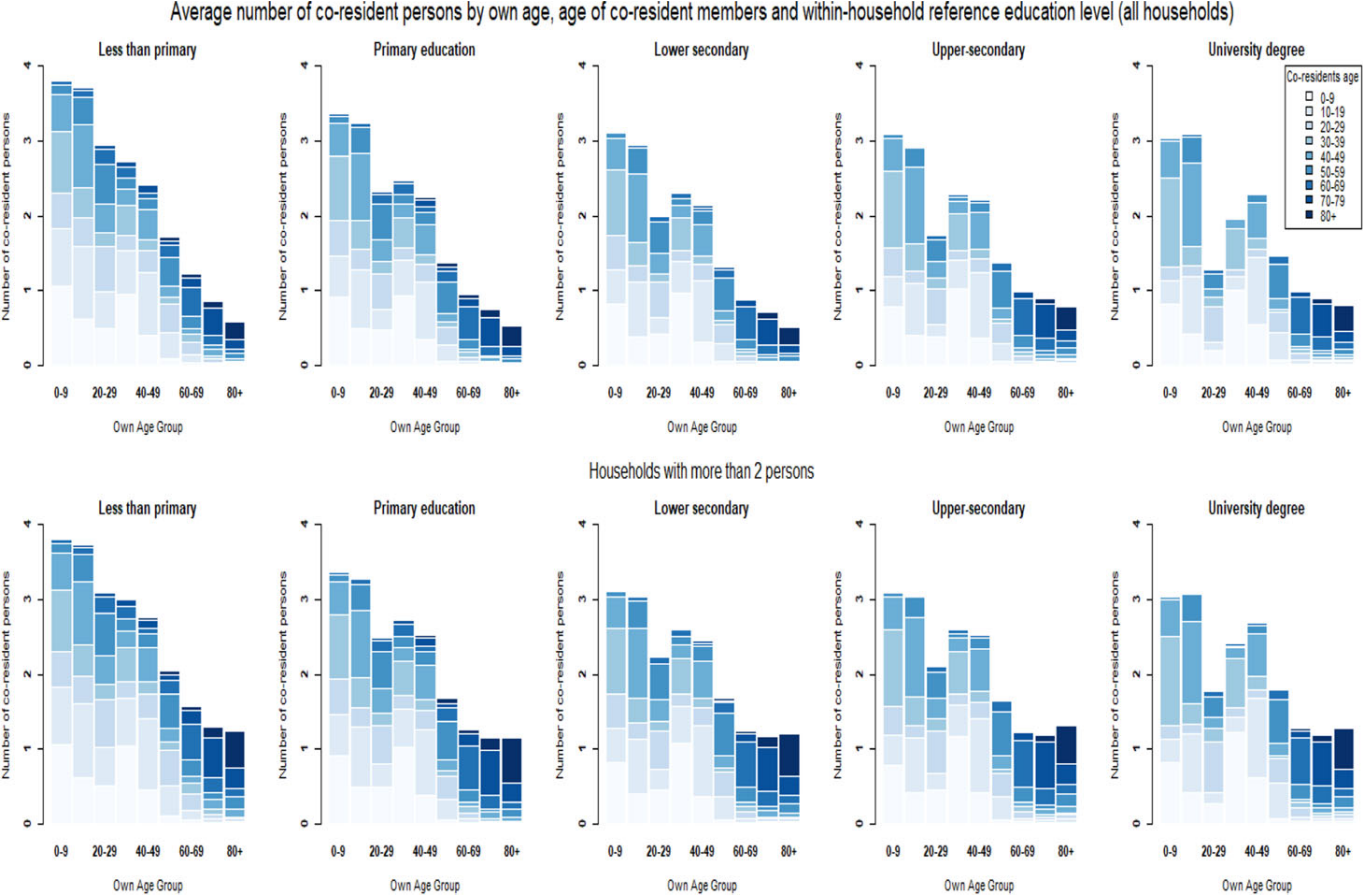
Household structures by head-of-household education level

## F. Results and robustness checks

### F.1 Sample restrictions

As a robustness test of our interpretations, the microsimulation exercise was conducted by restricting the sample in two different ways. First, by excluding persons in the sample living alone in order to be able to quantify the importance of single-households in the variations in indirect deaths observed between sub-populations. This test aims both to verify that the results observed are not biased by people living alone and to provide elements for assessing the explanations provided for the inequalities observed at different ages and the role that living alone may play depending on the categorical variable studied (Fig. 15, 16, and 17). The results were replicated for fixed household sizes to provide additional insights to our interpretations (Tables 13, 14, and 15).

**Fig. 9:**
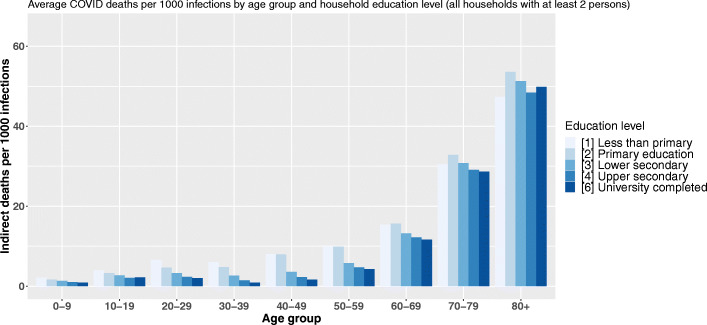
Average COVID-19 deaths per 1000 infections by age group and head-of-household education level (2+ members)

**Fig. 10:**
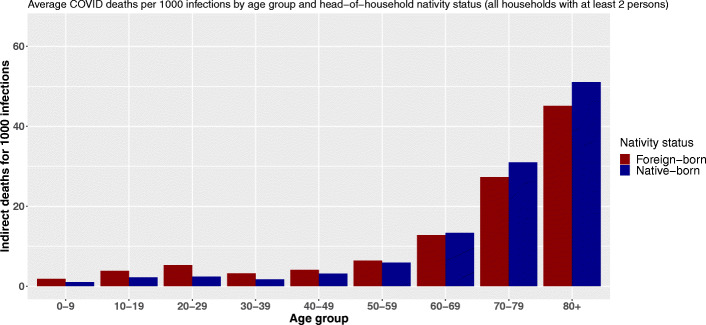
Average COVID-19 deaths per 1000 infections by age group and head-of-household nativity status (2+ members)

**Fig. 11:**
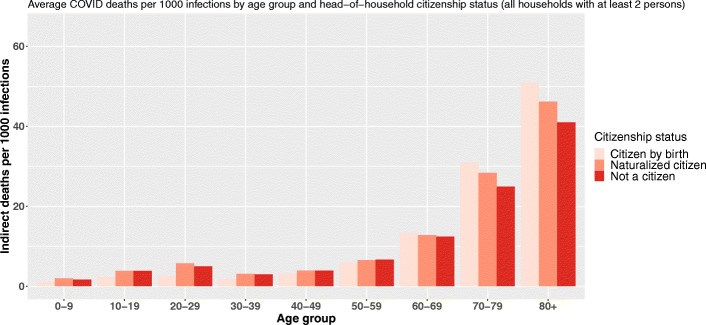
Average COVID-19 deaths per 1000 infections by age group and head-of-household citizenship status (2+ members)

**Table 13:** Theoretical over/under-mortality ratio related to secondary infections (head of household nativity status-%)

	Foreign-born					
	**Household type**					
**Age**	Full sample	2+ members	2 members	3 members	4 members	5 members
0–9	+83.40	+83.40	+46.40	+41.20	+51.00	+35.10
10–19	+79.60	+76.60	+42.00	+53.20	+69.30	+60.40
20–29	+141.30	+118.40	+73.50	+61.40	+73.50	+51.20
30–39	+95.40	+88.80	+33.60	+70.10	+143.10	+81.00
40–49	+35.00	+30.40	+13.90	+34.00	56.60	+22.80
50–59	+12.60	+8.00	+10.60	+5.00	+8.10	− 19.00
60–69	− 0.40	− 4.50	+2.80	− 18.80	− 27.60	− 43.10
70–79	− 6.30	− 12.00	− 6.80	− 21.50	− 33.30	− 33.90
80+	− 2.40	− 11.60	− 8.70	− 9.80	− 10.90	− 31.70

Reference level: native-born

*Note*: In the full sample, the primary infection of a 0–9-year-old living in a household headed by a foreign-born persons triggers a vulnerability index 83.4% higher than for the primary infection of a person of the same age living in a household headed by a person from the reference category

**Table 14:** Theoretical over/under-mortality ratio related to secondary infections (head-of-household citizenship status-%)

	Household type					
	**Full sample**		**2+ members**		**4 members**	
Age	Naturalized citizen	Not a citizen	Naturalized citizen	Not a citizen	Naturalized citizen	Not a citizen
0–9	+93.20	+60.70	+93.20	+60.70	+42.40	+30.20
10–19	+73.50	+72.30	+69.60	+69.90	+57.50	+56.70
20–29	+157.60	+109.50	+123.80	+93.70	+67.00	+58.60
30–39	+83.50	+74.20	+75.30	+68.70	+95.80	+131.80
40–49	+29.00	+26.80	+22.60	+23.10	+39.10	+52.80
50–59	+18.00	+19.20	+9.80	+12.60	+8.80	+16.10
60–69	+2.30	+0.60	− 4.20	− 6.90	− 29.20	− 28.40
70–79	− 1.00	− 9.40	− 8.40	− 19.30	− 32.30	− 36.20
80+	− 0.90	1.80	− 9.20	− 19.50	− 11.30	− 14.50

Reference level: citizen by birth

*Note*: When the full sample is considered, the primary infection of a 0–9-year-old living in a household headed by a naturalized citizen triggers a vulnerability index 58.7% higher than for the primary infection of a person of the same age living in a household headed by a person from the reference category

**Table 15:** Theoretical over/under-mortality ratio related to secondary infections (head-of-household education level, %)

	Household type											
	**Full sample**				**2+ members**				**4 members**			
Age	[1]	[2]	[3]	[4]	[1]	[2]	[3]	[4]	[1]	[2]	[3]	[4]
0–9	+130.50	+78.10	+43.80	+13.00	+130.50	+78.10	+43.80	+13.00	+130.50	+78.10	+43.80	+13.00
10–19	+77.50	+45.80	+18.00	− 9.00	+77.40	+46.40	+20.60	− 5.40	+77.40	+46.40	+20.60	− 5.40
20–29	+337.90	+199.40	+102.00	+33.00	+228.60	+130.40	+61.50	+15.30	+228.60	+130.40	+61.50	+15.30
30–39	+646.80	+493.30	+221.60	+78.40	+579.30	+440.00	+199.90	+67.80	+579.30	440.00	199.90	+67.80
40–49	+405.00	+407.20	+124.80	+41.60	+393.50	+391.00	+123.00	+39.20	+393.50	391.00	+123.00	+39.20
50–59	+138.60	+131.80	+32.20	+13.80	+133.30	+133.00	+36.20	+10.90	+133.30	133.00	+36.20	+10.90
60–69	+32.10	+29.10	+4.80	+8.50	+32.70	+34.90	+13.50	+5.10	+32.70	+34.90	+13.50	+5.10
70–79	− 6.30	+0.10	− 12.70	+2.20	+6.40	+14.70	+7.50	+1.40	+6.40	+14.70	+7.50	+1.40
80+	− 30.50	− 20.60	− 31.20	− 7.30	− 5.00	+7.60	+2.90	− 2.80	− 5.00	+7.60	+2.90	− 2.80

Education nomenclature: [1] = less than primary, [2] = primary, [3] = lower secondary, [4] = upper secondary

Reference level: university degree

*Note*:When the full sample is considered, the primary infection of a 0–9-year-old living in a household headed by a person with less than primary education ([1]) triggers a vulnerability index 130.5% higher than for the primary infection of a person of the same age living in a household headed by a person from the reference category

### F.2 Setting all transmission rates to 1

**Fig. 12:**
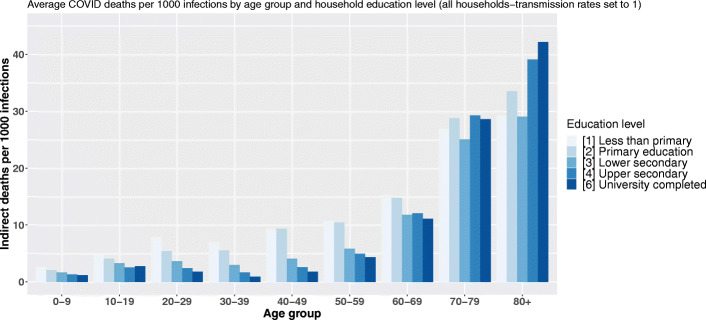
Average COVID-19 deaths per 1000 infections by age group and head-of-household education level (rates of transmission set to 1)

**Fig. 13:**
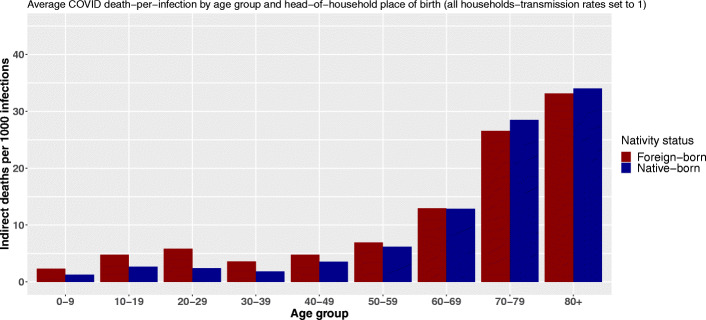
Average COVID-19 deaths per 1000 infections by age group and head-of-household nativity status (rates of transmission set to 1)

**Fig. 14:**
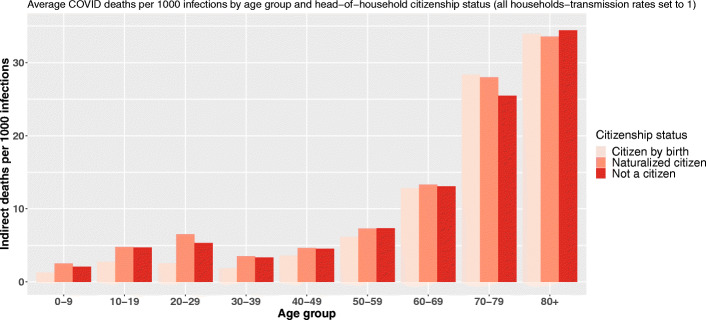
Average COVID-19 deaths per 1000 infections by age group and head-of-household citizenship status (rates of transmission set to 1)

### F.3 Alternative age-specific fatality ratios

**Fig. 15:**
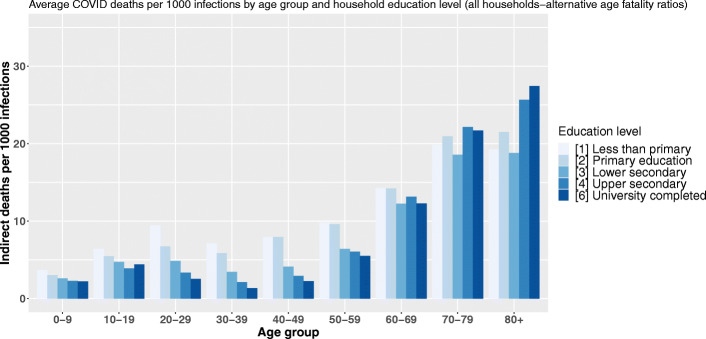
Average COVID-19 deaths per 1000 infections by age group and head-of-household education level (fatality ratios from Verity et al.)

**Fig. 16:**
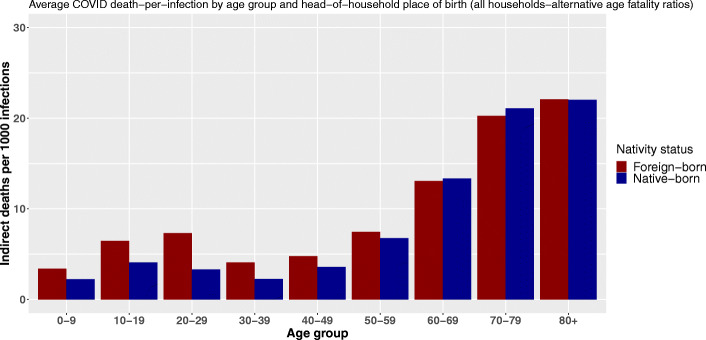
Average COVID-19 deaths per 1000 infections by age group and head-of-household nativity status (fatality ratios from Verity et al.)

**Fig. 17:**
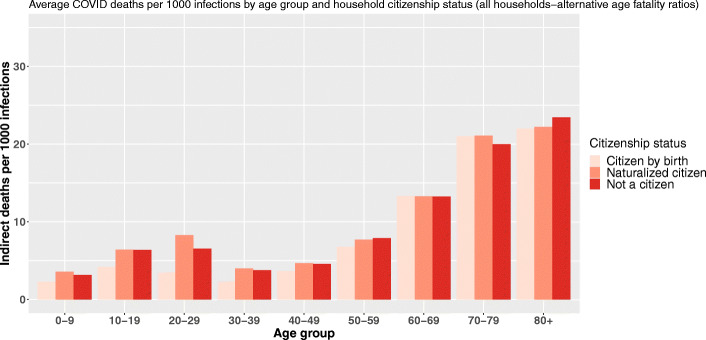
Average COVID-19 deaths per 1000 infections by age group and head-of-household citizenship status (fatality ratios from Verity et al.)

### F.4 Econometric model

A parametric estimation model is used to control for the effect of a set of variables on simulated indirect deaths following an infection. The model used is an ordinary least squares model with categorical variables interaction terms. The variable explained is the individual number of theoretical indirect deaths from our micro-simulation models. This model seeks to test whether the effects of education level and nativity status put forward in our analysis are robust to a parametric estimation controlling for a set of other socio-demographic factors. These variables are sex, region of residence, age, and urban/rural character of the area of residence. Beside these variables, the head-of-household education level and nativity status are included, and the estimates associated to these variables are used to compute their estimated effect on simulated indirect deaths. To compute the effects for both head-of-household education level and nativity status, three models were carried out. The first one includes either the interaction between age and education level or between age and nativity status, along the set of other socio-demographic variables. The second model includes the same set of variables and both interaction terms between age and head-of-household education level and age and head-of-household nativity status to control simultaneously for both of those variables. Formally, model 2 equation was:

$$\begin{array}{@{}rcl@{}} y_{i}&=&\beta_{0}+\beta X_{i}+\theta age_{i}*HHeducation_{i} + \gamma age_{i}*HHbirthplace_{i} +\epsilon_{i} \end{array} $$

The third model replicates the second one on the sub-sample of households with at least two persons.

All coefficients are significant at a 1% level. All estimation results are presented in the tables below in terms of differences in the number of deaths per 100,000 infections compared to a reference level.

**Table 16:** Parametric estimates results of COVID-19 mortality inequalities (head-of-household nativity status effect)

	Nativity status estimated mortality differences per 100,000 primary infections		
	Parametrical model		
Age	Without education control	With education control	With education control (2+ members)
0–9	+103	+81	+67
10–19	+186	+149	+136
20–29	+288	+193	+191
30–39	+158	+67	+52
40–49	+112	+16	− 15
50–59	+73	+2	− 47
60–69	+2	− 38	− 124
70–79	− 134	− 110	− 370
80+	− 63	− 22	− 516

Reference level: native-born

*Note*: In the model with education control, the random primary infections of 100,000 persons aged 0–9 living in household headed by a foreign-born person are associated with an estimated additional 81 deaths compared to 100,000 infections of individual of the same age and with similar socio-demographic characteristics but living in household headed by native-born persons

**Table 17:** Parametric estimates results of COVID-19 mortality inequalities (head-of-household education level effect)

	Education level estimated mortality differences per 100,000 primary infections											
	Parametrical model											
	Without nativity control				With nativity control				With nativity control (2+ members)			
Age	[2]	[3]	[4]	[5]	[2]	[3]	[4]	[5]	[2]	[3]	[4]	[5]
0–9	− 49	− 82	− 114	− 120	− 37	− 67	− 90	− 94	− 37	− 63	− 88	− 99
10–19	− 71	− 135	− 196	− 171	− 43	− 96	− 150	− 129	− 43	− 93	− 144	− 135
20–29	− 200	− 341	− 438	− 476	− 154	− 278	− 368	− 408	− 152	− 275	− 360	− 395
30–39	− 115	− 315	− 422	− 472	− 105	− 299	− 404	− 452	− 114	− 326	− 439	− 497
40–49	+2	− 391	− 510	− -561	+4	− 388	− 508	− 557	− 6	− 447	− 578	− 640
50–59	− 24	− 373	− 438	− 480	− 25	− 373	− 439	− 478	− 12	− 428	− 532	− 576
60–69	− 28	− 249	− 217	− 290	− 37	− 258	− 337	− 296	− 8	− 260	− 354	− 406
70–79	+140	− 132	+183	+136	+123	− 144	+170	+133	+174	− 23	− 197	− 211
80+	+316	− 7	+733	+958	+314	− 7	+734	+962	+570	+349	+61	+232

Education nomenclature: [1] = less than primary, [2] = primary, [3] = lower secondary, [4] = upper secondary, [5] = university degree

Reference level: Less than primary education

*Note*: In the model with nativity control, the random primary infections of 100,000 persons aged 0–9 living in household headed by an individual with a lower secondary education ([3]) are associated with an estimated fewer 67 deaths compared to 100,000 infections of individual of the same age and with similar socio-demographic characteristics but living in household headed by a person with less than primary education (reference level).

## G. Household structures and number of couples over 80

**Table 18:** Number of couples in the household by head-of-household characteristics (at least one old person)

	Number of couples			
**Nativity status**	No married couples	1 couple	2 couples	3 couples or more
Foreign-born	54.60	44.50	0.80	0.00
Native-born	58.30	41.30	0.30	0.00
**Citizenship status**	No married couples	1 couple	2 couples	3 couples or more
Citizen by birth	58.40	41.30	0.30	0.00
Naturalized citizen	54.90	44.10	0.90	0.00
Not a citizen	47.80	50.90	1.20	0.00
**Education Level**	No married couples	1 couple	2 couples	3 couples or more
Less than primary	63.90	35.80	0.30	0.00
Intermediate level of education	61.40	38.40	0.20	0.00
Upper secondary	47.90	51.30	0.80	0.00
University completed	44.70	54.60	0.70	0.00

**Table 19:** Households distribution by number of couples and household size (nativity status)

	Head of household nativity status									
	**Foreign-born**					**Native-born**				
	NUMBER OF PERSONS LIVING IN THE HOUSEHOLD									
	1	2	3	4	5+	1	2	3	4	5+
No couple	82.90	13.30	2.60	0.80	0.40	86.50	11.20	1.70	0.40	0.10
1 couple	0.00	80.20	12.30	3.80	3.50	0.00	87.70	8.90	2.00	1.30
2 couples	0.00	0.00	0.00	35.90	64.00	0.00	0.00	0.00	44.10	56.00
3 couples	0.00	0.00	0.00	0.00	99.90	0.00	0.00	0.00	0.00	100.00

Scope: All households with at least of individual over 80 years of age

*Note*: 82.9% of households with at least one person over 80 years of age, headed by a foreign-born person and hosting zero couple are made up of a single person

**Table 20:** Households distribution by number of couples and household size (education level)

	Head of household education level																			
	**Less than primary**					**Intermediate education**					**Upper secondary education**					**University completed**				
	Number of persons living in the household																			
	1	2	3	4	5+	1	2	3	4	5+	1	2	3	4	5+	1	2	3	4	5+
No couple	84.50	12.80	2.00	0.40	0.10	88.50	9.90	1.20	0.30	0.10	83.40	12.80	2.60	0.80	0.40	82.90	12.80	3.00	0.90	0.40
1 couple	0.00	84.60	11.00	2.60	1.80	0.00	90.20	7.70	1.40	0.80	0.00	83.40	10.80	3.00	2.70	0.00	86.30	8.80	2.60	2.30
2 couples	0.00	0.00	0.00	45.70	54.30	0.00	0.00	0.00	52.80	47.10	0.00	0.00	0.00	36.20	63.80	0.00	0.00	0.00	36.10	64.00
3 couples	0.00	0.00	0.00	0.00	99.90	0.00	0.00	0.00	0.00	100.00	0.00	0.00	0.00	0.00	100.00	0.00	0.00	0.00	0.00	100.10

Scope: All households with at least of individual over 80 years of age

*Note*: 84.5% households with at least one person over 80 years of age, headed by a person with a less than primary education and hosting zero couple are made up of a single person

**Table 21:** Households distribution by number of couples and household size (Citizenship status)

	HEAD OF HOUSEHOLD EDUCATION LEVEL														
	**Citizen by birth**					**Naturalized citizen**					**Not a citizen**				
	NUMBER OF PERSONS LIVING IN THE HOUSEHOLD														
	1	2	3	4	5+	1	2	3	4	5+	1	2	3	4	5+
No couple	86.40	11.20	1.70	0.40	0.10	83.50	12.80	2.30	0.90	0.40	78.00	16.40	3.70	1.10	0.90
1 couple	0.00	87.60	9.00	2.00	1.30	0.00	81.10	11.10	3.60	4.20	0.00	73.70	15.80	5.40	5.10
2 couples	0.00	0.00	0.00	44.60	55.40	0.00	0.00	0.00	30.00	69.90	0.00	0.00	0.00	34.60	65.30
3 couples	0.00	0.00	0.00	0.00	99.90	0.00	0.00	0.00	0.00	100.00	0.00	0.00	0.00	0.00	100.00

Scope: All households with at least of individual over 80 years of age

*Note*: 86.s% households with at least one person over 80 years of age, headed by a citizen by birth person and hosting zero couple are made up of a single person

## Data Availability

The datasets generated and analyzed in the study are available in the IPUMS International repository, available online. Epidemological data available on data.gouv were also used.

## Footnotes

1 See the John Hopkins University Coronavirus Resource Center.

2 Population density of inhabited neighborhoods, phenomena of spatial segregation, inequalities in access to housing.

3 Inability to stop going to work physically, (non)existence of sick leave.

4 Inequalities in access to health insurance, co-morbidity factors.

5 Coordinated by the Institut national de la statistique et des études économiques (INSEE), the Institut national d’études démographiques (INED), and the Direction de la Recherche, des Études, de l’Évaluation et des Statistiques (DREES).

6 Even if the effectiveness of lockdown measures and the limitation of the epidemic may depend on a vector of causes such as institutions, health behaviors, prevalence of co-mortality factors, climate, or compliance with social distancing measures.

7 Source: National Housing Survey 2013, INSEE, cited in Bodier et al. (2015). For more details, see also Marpsat and de Peretti (2009).

8 Considering that all individuals living with an infected person would also be infected allows to give an upper-bound limit to the theoretical average vulnerability indexes. This assumption being very strong, it is only used as a robustness test for the results of our main model.

9 The probabilities of infection by age category are 0–9, 40%; 10–19, 38%; 20–29, 79%; 30–39, 86%; 40–49, 80%; 50–59, 82%; 60–69, 88%; 70–79, 74%; and 80 and more, 74%.

10 The age-specific infection fatality ratios estimated by Verity et al. (2020) were also used as a robustness check for our analysis. In the main model, the fatality rates used are 0–9, 0.000922%; 10–19, 0.000958%; 20–29, 0.0058%; 30–39, 0.0163%; 40–49, 0.065%; 50–59, 0.24%; 60–69, 1.23%; 70–79, 4.29%; and 80 and more, 9.95%.

11 The data used to compute those rates are available at data.gouv.fr.

12 Detailed figures on the heterogeneity of household structures across selected socio-demographic categories are available in the Appendix.

13 The reference person of the household is determined automatically using a rule based on the number of persons in the household. If the household has only one person, this person is the reference person. If the household has two persons, if they are a couple and of different sex, the man is the reference person; otherwise, the reference person is the oldest active person, or if neither of them is active, the oldest person. If the household has three or more persons, the three oldest persons in the household are considered. If a couple is identified among them, the oldest working man of this couple, failing this the oldest working man of this couple, failing this the oldest working woman of this couple, failing this the oldest woman of this couple is the reference person; otherwise, the reference person is the oldest working person among them, or if none of the three oldest persons in the household is active, the oldest person among them.

14 The similarity of the distribution of the individual level variables with the statistics published by the INSEE was verified upstream of the analysis of the head-of-household variables. The distribution of the sample by head-of-household categories is also available in Appendix A.

15 All the results of the micro-simulation models are available in Appendix G.

16 Here being the one born on French territory and their co-resident members.

17 This analysis of co-housing with a partner at older ages is supported by a focus on the number of couples in households by socio-economic category. The results are available in Appendix G.
